# Human fallopian tube epithelial cells exhibit stemness features, self-renewal capacity, and Wnt-related organoid formation

**DOI:** 10.1186/s12929-019-0602-1

**Published:** 2020-02-08

**Authors:** Yu-Hsun Chang, Tang-Yuan Chu, Dah-Ching Ding

**Affiliations:** 1 Stem Cell Laboratory, Department of Research, Hualien Tzu Chi Hospital, Buddhist Tzu Chi Medical Foundation, Hualien, Taiwan; 2 Department of Pediatrics, Hualien Tzu Chi Hospital, Buddhist Tzu Chi Medical Foundation; Tzu Chi University, Hualien, Taiwan; 3 Department of Obstetrics and Gynecology, Hualien Tzu Chi General Hospital, Buddhist Tzu Chi Medical Foundation, Tzu Chi University, 707, Sec. 3, Chung-Yang Rd., Hualien, 970 Taiwan; 4 Department of Life Sciences, Tzu Chi University, Hualien, Taiwan; 5 Department of Gyecology and Obstetrics, School of Medicine, Tzu Chi University, Hualien, Taiwan

**Keywords:** Fallopian tube, Epithelium, Stem cells, Differentiation, Organoid

## Abstract

**Background:**

Fallopian tube epithelial cells (FTEC) were thought to be the origin of high-grade serous ovarian carcinoma (HGSOC). Knowledge of the stemness or initiating characteristics of FTEC is insufficient. Previously, we have characterized the stemness cell marker of FTEC, this study aims to further characterize the clonogenicity and spheroid features of FTEC.

**Methods:**

We successfully derived FTECs from the epithelial layer of the human fallopian tubes. We examined the morphology, proliferation rate, doubling time, and clonal growth of them. At passage 3, the sphere formations on gelatin-coated culture, suspension culture, and matrigel culture were observed, and the expression of LGR5, SSEA3, SSEA4, and other stemness markers was examined. Furthermore, tissue-reconstituted organoids from coculture of FTEC, fallopian stromal cells (FTMSC) and endothelial cells (HUVEC) were examined.

**Results:**

FTEC exhibited cuboidal cell morphology and maintained at a constant proliferation rate for up to nine passages (P9). FTEC could proliferate from a single cell with a clonogenic efficiency of 4%. Flow cytometry revealed expressions of normal stem cell markers (SSEA3, SSEA4, and LGR5) and cancer stem cell markers (CD24, CD44, CD117, ROR1, and CD133). FTEC formed spheres and colonies when cultured on low attach dish. In the presence of Matrigel, the stemness and colony formation activity were much enhanced. In co-culturing with FTMSC and HUVEC, FTEC could form organoids that could be blocked by Wnt inhibitor DKK1. Expressions of LGR5 and FOXJ1 expression were also decreased by adding DKK1.

**Conclusion:**

We demonstrated abundantly presence of stem cells in human FTECs which are efficient in forming colonies, spheres and organoids, relying on Wnt signaling. We also reported for the first time the generation of organoid from reconstitutied cell lineages in the tissue. This may provide a new model for studying the regneration and malignant transformation of the tubal epithelium.

## Background

Epithelial ovarian cancer (EOC) is the fifth cause of cancer death in women and high-grade serous carcinoma is the most prevalent and lethal histotype of EOC [1]. More than 220,000 new ovarian cancer patients occur in the world annually [2]. Most patients with ovarian cancer were found in late-stage disease and 5-year survival is only 30–40% [1]. The mortality rate of ovarian cancer has not changed since 1930 [3] due to the late detection of ovarian cancer. Therefore, early detection or effective interruption of the carcinogenesis of these cancers is important to resolve this dilemma. Ovarian cancer may develop from tissue stem cells [4].

Recently, the secretory cell lineage in the epithelium of  the fallopian tube especially in the fimbrial partis regarded as the main origin of high-grade serous ovarian carcinoma (HGSOC) [5, 6]. Transformation of the tubal epithelium seems to follw the clonal evolution model with step-wise accumulation of muttions and loss of progesterone clearance on the p53 initiated secretory known as  "p53 signature" and evenetually ends with serous tubal intraepithelial carcinoma and HGSC [7]. Although the secretory cell linage is regarded to be the origin of HGSOC, the stemness characteristic of the so called tumor initiation cell in the fallopian tube epithelium has not been well elucidated.

Epithelium of fallopian tube fimbria is regularly exposed to the follicular fluid during each ovulation [8]. The follicular fluid contains many abundant ROS and inflammatory cytokines which readily lead to tissue injury and DNA double strand breaks to the epithelium [8]. Thus the fallopian tube fimbria epithelium is one of the tissues with most abundant stemness activity [9]. Previoiusly, we have showed that exposure to the insulin-like growth factor axis proteins in the follicular fluid leads to stemness activation and clonal expansion of the human fimbrial epithelial cells [10]. However, characteristics of the stem-like cell (FTESC) especially the cell surface marker in this epithelium is still obscur [11–13]. The FTESCs express EpCAM, CD44, and integrin alpha 6 but did not express PAX8 and TUBB4 [11]. Yamamoto et al. derived FTESC from fallopian tube epithelial cells (FTEC) and proposed the stem cell markers of FTEC as PAX8+, FOXJ1-, and PAX2- [12]; Wang et al. found quiescent cell located at distal fallopian tube could be the origin of stem cells that could differentiate into different glandular cells [13]. Our previous study also showed expressions of stemness markers (SSEA3, SSEA4, and ALDH1) and stemness gene expressions (WNT and NOTCH signaling) in FTEC were affected by estradiol and progesterone [14].

The aims of this study were to derive FTECs from the distal fallopian tube and characterize them with morphology, proliferation, surface markers, gene expressions, and organoid formation.

## Materials and methods

### Derivation and culture of the primary fallopian tube epithelial cell (FTEC)

The experimental protocol was approved by the Research Ethics Committee of Hualien Tzu Chi Hospital (IRB 104-70B). Surgical specimens of fallopian tubes were obtained from premenopausal patients under a bilateral salpingectomy accompanied by hysterectomy or other ovarian surgery with benign indications. The fimbria part of the fallopian tube was used. For FTEC culture, we followed the previously published protocol with modification [11]. Briefly, the fimbria tissue was washed in 5 mM EDTA and incubated in 1% of trypsin for 45 min. Then the epithelium separated from the underlying stroma by digestion at 37 °C for 45 min with 0.8 mg/ml collagenase in DMEM supplemented with 10% FBS and 5 μg/ml insulin. The isolated epithelium was then incubated in prewarmed 0.05% trypsin-EDTA (Invitrogen, Grand Island, NY, USA). By using a 22 gauge needle, they were passed five times for dissociation and DMEM containing 10% FBS was added to stop the trypsinization. The resulted FTECs were grown on gelatin-coating wells in the following experiments.

### Proliferation assay and accumulated population doubling

The FTECs were seeded in triplicate at a density of 2 × 10^3^ cells/cm^2^, in a 96-well plate with DMEM with 10% FBS and 5 μg/ml insulin. FTEC at passage 2–3 were used for proliferation assay. Cells were harvested and counted using a cell proliferation kit (XTT based, Biological Industries Ltd., Kibbutz Beit Haemek, Israel) on days 0, 3 and 7, and a growth curve was generated. XTT solutions and PMS (N-methyl dibenzopyrazine methyl sulfate) were defrosted immediately prior to use in a 37 °C bath. PMS was added to the XTT solution immediately before use. 50 μl of XTT/PMS was added to each 100 μl culture. After 2–5 h of incubation, the optical density (OD) of the wells was determined using a spectrophotometer (ELISA reader) at a wavelength of 450 nm and a reference wavelength of 650 nm.

To calculate the population doubling time (DT), 1 × 10^4^ cells were seeded in a 10-cm Petri dish. The culture medium was changed on day 4 and the cells were harvested and counted on day 7. The DT was calculated according to the formula: DT = log (final cell number) - log (initial cell number) = K × T, where K is the generation constant (0.008963) and T is the time in hours [15].

### Flow cytometry of FTEC

Surface molecules of cultured FTEC of passage 2, 3, and 8 were characterized by a flow cytometer (Becton Dickinson, San Jose, CA, USA). Three FTEC cell lines were enrolled for testing for LGR5, SSEA3, and SSEA4. Cells were detached with 2 mM EDTA in PBS, washed with PBS containing 2% bovine serum albumin (BSA) and 0.1% sodium azide (Sigma, USA), and incubated with their respective antibody conjugated with fluorescein isothiocyanate (FITC) or phycoerythrin (PE). The FTEC were identified by fallopian tube epithelial stem cell marker LGR5 [GeneTex, Irvine, CA, USA], and embryonic stem cell markers SSEA3 [eBioscience] and SSEA4 [Abcam]. The cancer stem cell markers including CD24 (eBioscience), CD44 (eBioscience), CD117 (eBioscience), ROR1 (BD) [16], and CD133 (eBioscience) [17] were also characterized in FTEC and control HGSOC cell lines. HGSOC cell lines (KURAMOCHI and OVSAHO) were used as positive controls [18].

We assayed the ALDH activity by using the Aldefluor assay kit (Stem Cell Technologies, Inc., Cambridge, MA, USA). We trypsinized FTECs and incubated with activated ALDEFLUOR reagent at 37 °C for 50 min. The ALDH inhibitor DEAB was added to differentiate ALDH(−) and ALDH(+) subpopulations. All stained cells were subjected for analysis by using a flow cytometer. For sorting subpopulations of ALDH+ and ALDH-, the FTECs were collected using a BD FACSAaria II cell sorter (BD Biosciences).

### KURAMOCHI and OVSAHO cell culture

HGSOC cell lines KURAMOCHI and OVSAHO were obtained from the Japan cell bank. Both cell lines were reported as HGSOC cell lines according to their gene expression patterns [19]. These cells were grown as monolayers at 37 °C in a humidified atmosphere with 5% CO2 in RPMI 1640 medium (Gibco) supplemented with 10% (v/v) FBS, penicillin (50 U/ml) and streptomycin (50 μg/ml).

### RNA extraction and quantitation

Total RNA of P3 FTECs after culturing on gelatin or matrigel for 7 days was extracted using an RNEasy® kit (Qiagen, Valencia, CA, USA) according to the manufacturer’s instructions.

For quantitative RT-PCR analysis of FTEC stem cell marker, the primer sequences were as follows: LGR5, (primer sequence: forward: 5′- GCAAACCTACGTCTGGACAA-3′; 5′- TGATGCTGGAGCTGGTAAAG-3′), SSEA4 synthase (forward: 5′- TGGACGGGCACAACTTCATC-3′; reverse: 5′- GGGCAGGTTCTTGGCACTCT-3′); b3GalT5 (SSEA3) (forward: 5′- GCAGATCTATGGCTTTCCCGAAGATG-3′; reverse: 5′- GTCTCGAGTCAGACA GGCGGACAAT-3′); ALDH1 (forward: 5′-TTG GAA TTT CCC GTT GGT TA-3′; reverse: 5′-CTG TAG GCC CAT AAC CAG GA-3′).

Real-time PCRs were performed and monitored using FastStart Universal SYBR Green Master (ROX, Roche, Indianapolis, IN, USA) and a quantitative real-time PCR detection system (ABI Step One Plus system, Applied Biosystems, Foster City, CA, USA). The gene products were analyzed with the *GAPDH* gene (forward: 5′-TCT CCT CTG ACT TCA ACA GCG AC-3′; reverse: 5′-CCC TGT TGC TGT AGC CAA ATT C-3′) as a reference. The expression level of each target gene was then calculated as 2^-ΔΔCt^, as previously described [20]. Four readings of each experimental sample were obtained for each gene of interest, and the experiments were repeated at least three times.

### Clonal growth assay

Prior to plating into low attach dish, FTECs were transfected with RFP (marked with a red fluorescent protein, ThermoFisher) and GFP (marked with a green fluorescent protein, Invitrogen) and mixed in growth. To assess the clonal growth of FTEC, we considered the single color sphere as the colony derived from one single cell.

### Suspension sphere formation

The FTECs were cultured in 6-well with the non-adhesive surface (Corning, Corning, NY, USA) [21]. Cells were plated at a density of 5 × 10^4^ cells/well, with the serum-free DMEM/F12 supplemented with 5 μg/ml insulin, 20 ng/ml human recombinant epidermal growth factor (EGF; Invitrogen), 10 ng/ml basic fibroblast growth factor (bFGF; Invitrogen), and 0.4% bovine serum albumin (BSA; Sigma); these media were changed every other day for 14 days. The resulted spheres were then fixed and stained of LGR5 with immunohistochemistry.

### Colony formation of FTEC cultured on Matrigel

The subpopulations of ALDH+ and ALDH- FTECs were collected by sorting described above. 25,000 ALDH+ or ALDH- FTECs per well were cultured in 6 well dishes pre-coated with 50 μl 1% Matrigel (BD Matrigel Basement Membrane Matrix) at 37 °C in a 5% CO_2_ atmosphere. The matrigel was solidified for 20 min at 37 °C and overlaid with 500 μl culture medium (DMEM supplemented with 10% FBS and 5 μg/ml insulin). Colonies were counted after 14 days. If the diameter of the colony more than 100 μm, we classified as large spheres. The colonies’ diameter from 10 to 100 μm were classified as small colonies.

### Matrigel organoid culture

For organoid culture, 25,000 FTECs were cultured in 6 well dishes pre-coated with Matrigel (50 μl of 1% Matrigel (BD Matrigel Basement Membrane Matrix)) at 37 °C in a 5% CO2 atmosphere and overlaid with 500 μl organoid culture medium. The organoid culture medium consisted of DMEM supplemented with 50 ng/ml Wnt3a, 50 ng/ml RSPO1 (R & D, Minneapolis, MN, USA), 12 mM HEPES, 1% glutamax, 2% B27, 1% N2, 10 ng/ml EGF (Invitrogen), 100 ng/ml noggin, 100 ng/ml FGF10 (Peprotech), 1 mM nicotinamide, 9 μM ROCK inhibitor (Y-27632, Sigma), and 0.5 μM TGF-β R kinase inhibitor IV (SB431542, Sigma). After more than 21 days of culture, organoids were sent to immunohistochemistry for FOXJ1, detyrosinated TUBULIN (ciliated cell markers), PAX8 (a secretory cell marker), and vimentin (a mesoderm marker).

### Three-combined organoid culture with FTEC, mesenchymal stem cells (FTMSC) and human umbilical vein endothelial cells (HUVEC)

FTMSCs were derived from FT stroma after removing the epithelial layer from human FT fimbria. After washed in 5 mM EDTA and incubated in 1% of trypsin for 45 min, FT stroma was digested in 0.8 mg/ml collagenase in DMEM supplemented with 10% FBS and 5 μg/ml insulin at 37 °C for 45 min. It was then incubated in prewarmed 0.05% trypsin-EDTA (Invitrogen, Grand Island, NY, USA), passed five times for dissociation using a 22-gauge needle, and added with DMEM medium containing 10% FBS. The resulted FTMSCs were plated in 10-cm dishes. After proliferation to 80% confluent, the cells were passed with a 1:3 ratio. The HUVECs (BCRC, Hsinchu, Taiwan) were cultured in Endothelial Cell Medium (Promocell, Biochief International Co. Ltd., Taipei, Taiwan) and changed medium every 2–3 days.

The three-combined organoid culture was done by mixed FTEC, FTMSC, and HUVEC in a ratio of 10:7:2. A total of 1 × 10^6^ cells were cultured in one of the 24-well plates pre-coating with Matrigel as previously described [22]. The culture medium consisted of the organoid culture medium for FTEC and two regular media for FTMSC and HUVEC, mixed with the same ratio. In the inhibition assay, we treated with the three-combined culture with DKK1 (R & D system) at 250 ng/ml for 48 h. DKK1 is a natural inhibitor for Wnt signaling [23]. The medium was changed every other day and the organoid formation was observed after 7 days.

### Immunofluorescence staining

Spheres and organoids were fixed in 4% of paraformaldehyde for 1 h at room temperature. Alcohol was used for a dehydrated purpose and followed by acetone and isopropanol for 20 min at room temperature. The dehydrated spheres and organoids were embedded in the paraffin block and sectioned with a 5-μm thickness. Immunofluorescence staining was carried out with rehydrated sections and treated with antigen retrieval solution (Dako). Then we blocked the sections with 1% BSA and 2% FCS in PBS for 1 h. Then the sections were incubated with primary antibodies diluted 1:100 for 1.5 h. Then we washed the sections with PBS with 0.1% Tween 20 and incubated with secondary antibodies for 1 h. Finally, the sections were washed with PBS with 0.1% Tween 20 and mounted in Antifade solution (Sigma-Aldrich).

### Immunohistochemistry (IHC)

In IHC, we used anti-PAX8 antibodies for secretory cells, (1:50, [ab53490, Abcam]), anti-TUBB4 for ciliated cells and EpCAM for epithelial cells, LGR5 for stem cells, and DAPI for the nucleus (1:200, GeneTex, Irvine CA, USA). Briefly, paraffin sections were de-paraffinized, rehydrated, and treated with 10 mM citrate buffer at 95 °C to retrieve antigens, then blocked with 5% BSA and incubated with the above-mentioned antibodies overnight. The tissue sections were then incubated with secondary antibodies and DAB reagent (Abcam). The sections were then counterstained with hematoxylin, dehydrated, and visualized with 3.3-diaminobenzidine (Abcam). The stained slides were imaged on a Zeiss Axiovert with EXFO X-Cite series 120Q source, Zeiss McR camera, with Axiovision software (triple-stained samples) (Zeiss, Thornwood, NJ, USA).

### Statistical analysis

The results were expressed as the mean ± standard deviation (SD). Raw data were analyzed using Student t-test for comparison of gene expressions between two culture methods, where a *p-value* < 0.05 denotes statistical significance. SPSS software was used for this analysis (version 20, IBM, NY, USA).

## Results

### Human fallopian tube expressed PAX8, TUBB4, EpCAM and LGR5

**Fig. 1:**
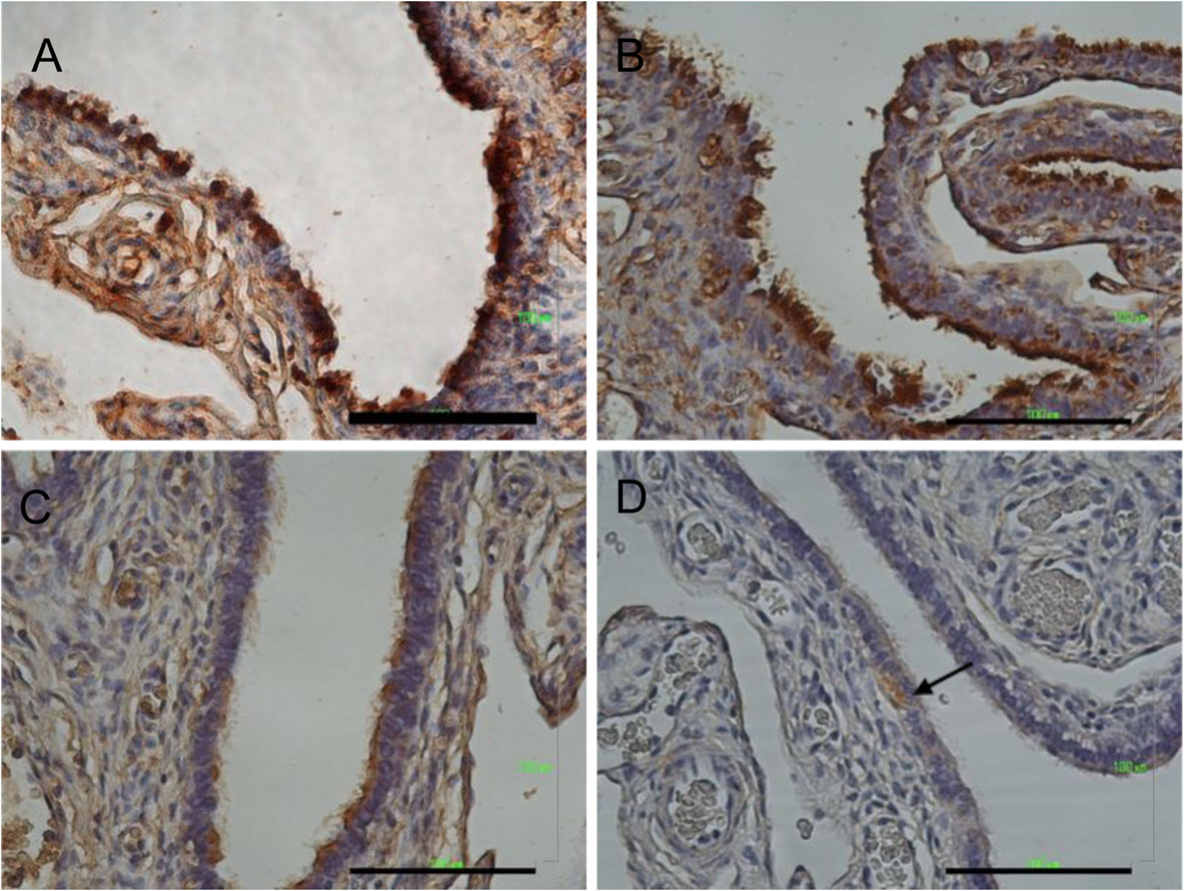
Immunohistochemistry of fallopian tube tissue specimen. (**a**) PAX8 marked the secretory cells. (**b**) TUBB4 marked the ciliated cells. (**c**) EpCAM marked epithelial cell lineage. (**d**) LGR5 marked the stem cells (arrow). Scale bar = 100 μm

The expression of epithelial and stem cell markers was examined in the human fallopian tube fimbria epithelium. The FT specimen expressed extensively PAX8 (secretory cell marker), TUBB4 (ciliated cell marker), and EpCAM (epithelial cell marker) (Fig.1a-c). Few cells expressed LGR5 (stem cells marker) (Fig. 1d).

### FTEC isolated from epithelial layer grew up to passage 9 and expressed various cancer stem cell markers

**Fig. 2:**
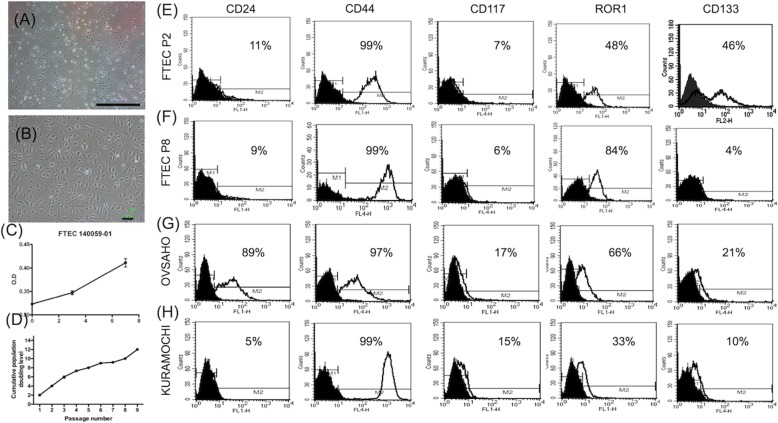
The morphology and growth kinetics of fallopian tube epithelial stem cells (FTEC). (**a, b**) After dissociation and culture, FTEC showed polygonal morphology at P2 (**a**) and P6 (**b**). Scale bar 100 μm. (**c**) After culturing for 7 days, the growth kinetics of FTEC using XTT assay was evaluated on day 3 and day 7. (**d**) Cumulative population doubling of FTEC. Population doubling was measured at each passage. Data are expressed as the mean ± SD (*n* = 3). (**e-h**) Flow cytometry analysis for the expression of cancer stem cell markers on FTEC and HGSOC cell lines. CD24, CD44, CD117, ROR1, and CD133 were evaluated in low (P2, **e**), high passages (P8, **f**) of FTEC, and in KURAMOCHI (**g**) and OVSAHO (**h**) cells

We isolated and cultured FTEC from the FTE sheet. FTEC at P2 (Fig. 2a) and P6 (Fig. 2b) showed the same morphology with a cobblestone appearance. FTEC at P3 showed a normal cell growth pattern in 7 days (Fig. 2c). Passages from P1 to P9 could be accomplished in 50 days (Fig. 2d). A slowdown of proliferation occurred after P6 but recovered after P8 (Fig. 2D). We compared the expression of putative cancer stem cell markers (CD24, CD44, CD117, ROR1, and CD133) of HGSC [31134503] at P2 and P8, as well as two HGSOC cell lines. In FTEC, there was the same low expression of CD24 and high expression of CD44 and CD117 in P2 and P8. Compared to P2, P8 FTEC had a higher (85% vs. 48%) expression of ROR1 and lower (4% vs. 46%) expression of CD133 (Fig. 2e). Among the two HGSOC cells, KURAMOCHI showed cell surface markers pattern very similar to FTEC (other than a lower expression of CD133), whereas OVSAHO had a much lower CD24 and lower CD133 (Fig. 2f).

### FTECs expressed normal stem cell markers of LGR5, SSEA3, SSEA4, ALDH

**Fig. 3:**
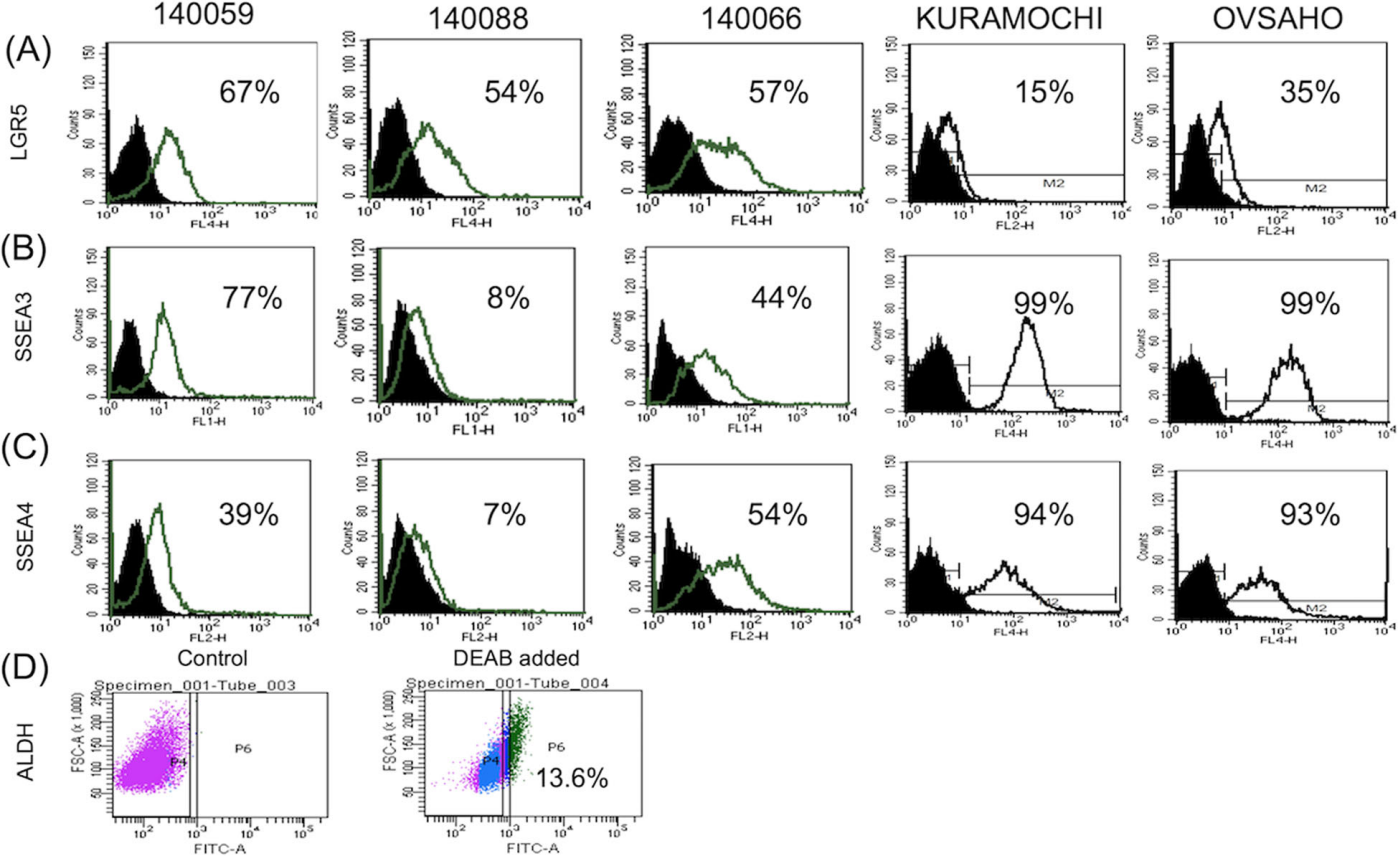
Flow cytometry analysis for the expression of normal stem cell markers on FTEC. (**a**-**c**) Flow cytometry illustrated expressions of LGR5(**a**), SSEA3 (**b**), and SSEA4 (**c**) in three FTEC cell lines at passage 3. HGSOC cell lines (KURAMOCHI and OVSAHO) were used as a positive control and IgG used as a negative control. (**d**) FACS analysis of the percentage of ALDH+ cell population in FTEC. FTECs owned 13.6% ALDH+ cells after DEAB treatment

Three stem cell markers, LGR5 (intestinal and fallopian tube stem cell marker), SSEA3 and 4 (embryonic and cancer stem cell marker), and ALDH were chosen for further evaluation. All 3 FTEC lines at P3 expressed these three stem cell markers with different percentages (Fig. 3). All three FTEC cell lines expressed LGR5 in > 50% of the population. One cell line showed less expressed of SSEA3 (8%) and SSEA4 (7%), the other two expressed 40% or more of the two markers. On the contrary, the two HGSOC cell lines, KURAMOCHI and OVSAHO, less expressed LGR5 (15 and 35%, respectively) and highly expressed SSEA3 and SSEA4 (> 93%). We also sorted ALDH(+) and ALDH(−) cells from FTEC. As shown in Fig. 3d, 13.6% of FTECs were ALDH(+). These results inferred that isolated FTECs had a large population of stem cells expressing stem cell markers including LGR5, CD44, ROR1, and CD133.

### Sphere formation and clonal growth of FTEC

**Fig. 4:**
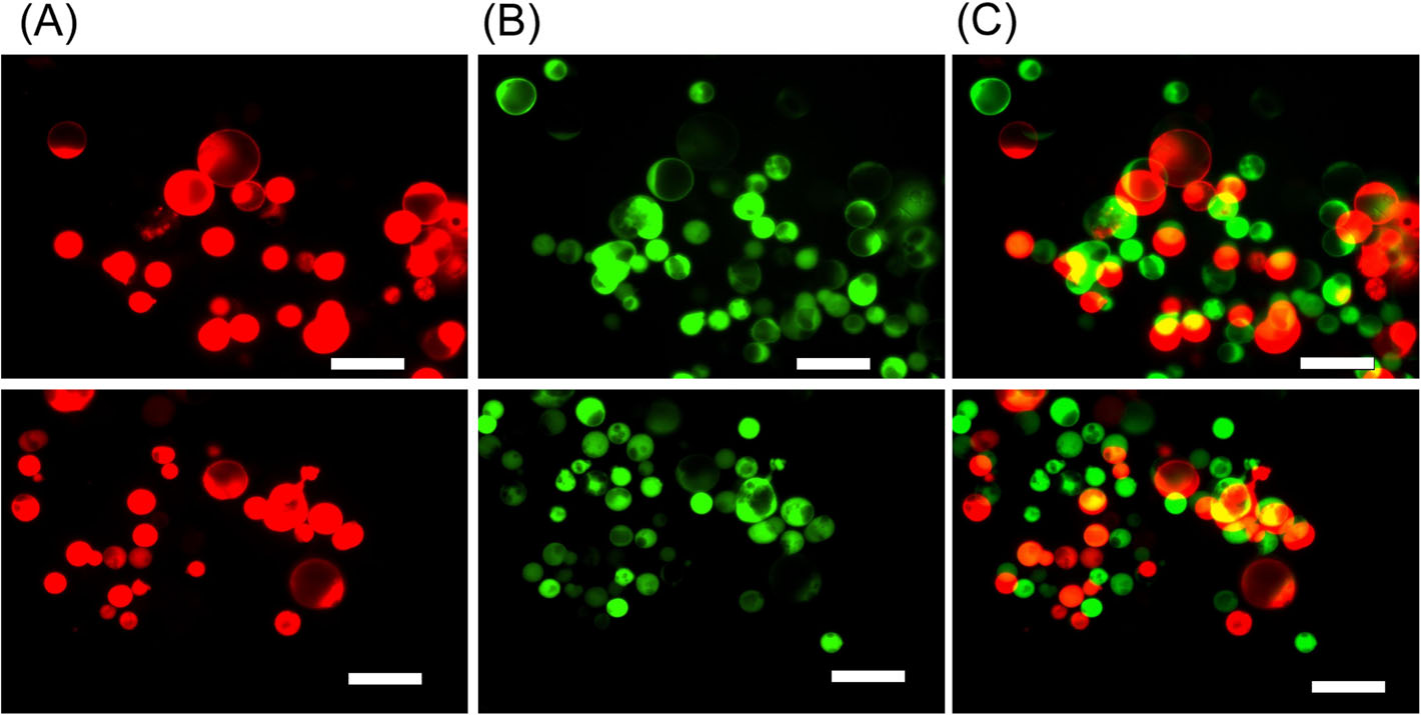
The colonial growth of FTEC on Day 5**.** FTECs were transfected with RFP (marked with red fluorescent protein) and GFP (marked with green fluorescent protein). Both FTECs were mixed prior to plating into the low attached dish. Regenerated spheres (colonies) were either positive for RFP or GFP. (**a**) RFP. (**b**) GFP. (**c**) Merge image

To know whether single FTEC could generate one sphere (colony), FTECs were transfected with RFP (marked with red fluorescent protein) or GFP (marked with green fluorescent protein). Both were mixed prior plating into low- attachment dish. Regenerated spheres were either positive for RFP or GFP (Fig. 4). The chimeric sphere was not detected. The result suggested FTE had sphere-forming activity and the spheres were derived from a single cell.

### FTEC sphere showed the feature of fallopian tube epithelium

**Fig. 5:**
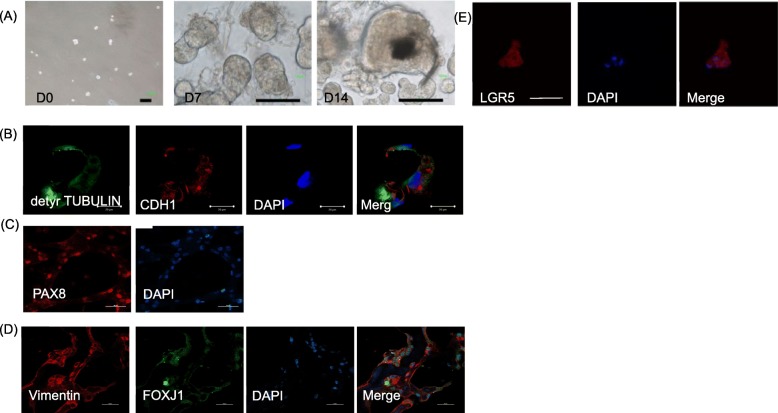
FTEC formed spheres had structures, markers, and stem cell markers similar to those in the tubal epithelium. (**a**) FTEC cultured in a low-attachment dish in serum replacement medium from Day 0 to Day 14. The increasing size was noted at 7 days and 14 days. (**b-e**) Confocal immunofluorescence image of tubal epithelial cell markers including detyrosinated TUBULIN and CDH1 (an epithelial cell marker), PAX8 (red) and DAPI (blue, nuclei), vimentin (red) and another ciliated cell marker FOXJ1 (green). Scale bar = 50 μm. (**e**) The FTEC spheroids revealed positive IHC for LGR5. DAPI (blue) staining represented cell nuclei. Scale bar = 50 μm

After 14 days of low-attachment suspension culture, 5 × 10^4^ FTEC formed approximately 2 × 10^3^ spheres in serum-free medium supplemented with EGF, bFGF, and insulin (Fig. 5a). The efficiency of sphere formation was 4%. The spheres in serum replacement suspension culture showed the architectural features of fallopian tube epithelium, i.e. polarized columnar cells, aligned nuclei, and expressed main epithelial adhesion molecule E-cadherin (CDH1) at cell-cell junctions and Mullerian duct epithelium structure protein vimentin (Fig. 5b). Markers of the two fallopian tube epithelial cells, i.e. secretory cells (PAX8+) and ciliated cells (PAX8-, acetylated TUBULIN+, FOXJ1+) were demonstrated in the mature sphere (Fig. 5c). In addition, stem cell marker LGR5 was expressed in the majority of sphere cells (Fig. 5e). The results concluded FTEC in a suspension culture could form spheres that recaptured FTE architecture and express typical FTE cell markers. Moreover, LGR5 was also expressed in this suspension culture.

### ALDH+ FTEC formed more colonies on Matrigel

**Fig. 6:**
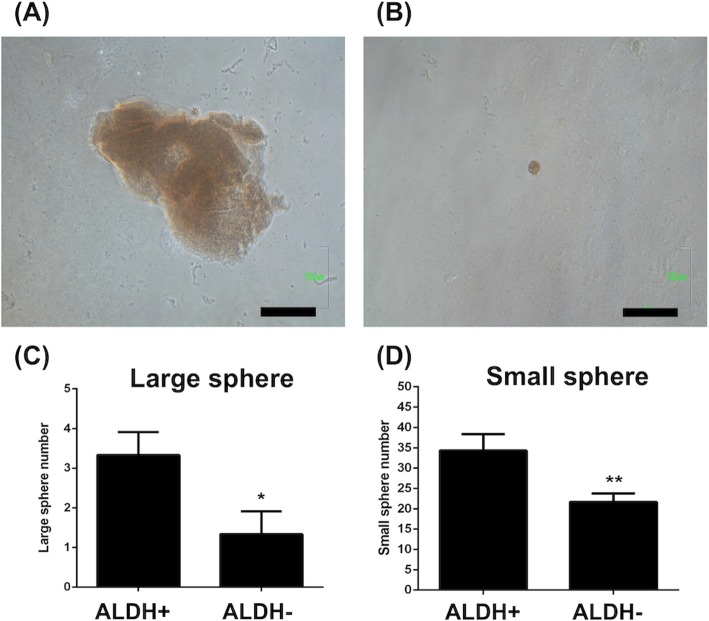
Colony formation of FTEC cultured on matrigel. FTEC formed a large colony (**a**) and small colony (**b**) on Matrigel culture. ALDH+ FTEC formed more large (**c**) and small (**d**) colony on Matrigel. *p < 0.05, **p < 0.01

The clonogenicity capacity of FTEC was characterized by colonies formation on Matrigel. As indicated in Fig. 3d, 13.6% of the FTEC population are ALDH+. We tested the clonogenic activity of FTEC in relation to the status of ALDH. In Matrigel-coated dish and regular culture medium with serum and insulin, FTEC could form large and small colonies (Fig. 6a-b). Large colonies were defined as the diameter of colonies more than 100 μm. As shown in Fig. 6c-d, ALDH+ cells formed more large and small colonies than ALDH- cells (3.3 ± 0.3 vs. 1.3 ± 0.3 / 2500 cells, *p* < 0.05; 34.3 ± 2.3 vs. 21.6 ± 1.2 / 2500 cells, *p* < 0.01, respectively).

### FTEC grown on Matrigel increased stem cell marker expression

**Fig. 7:**
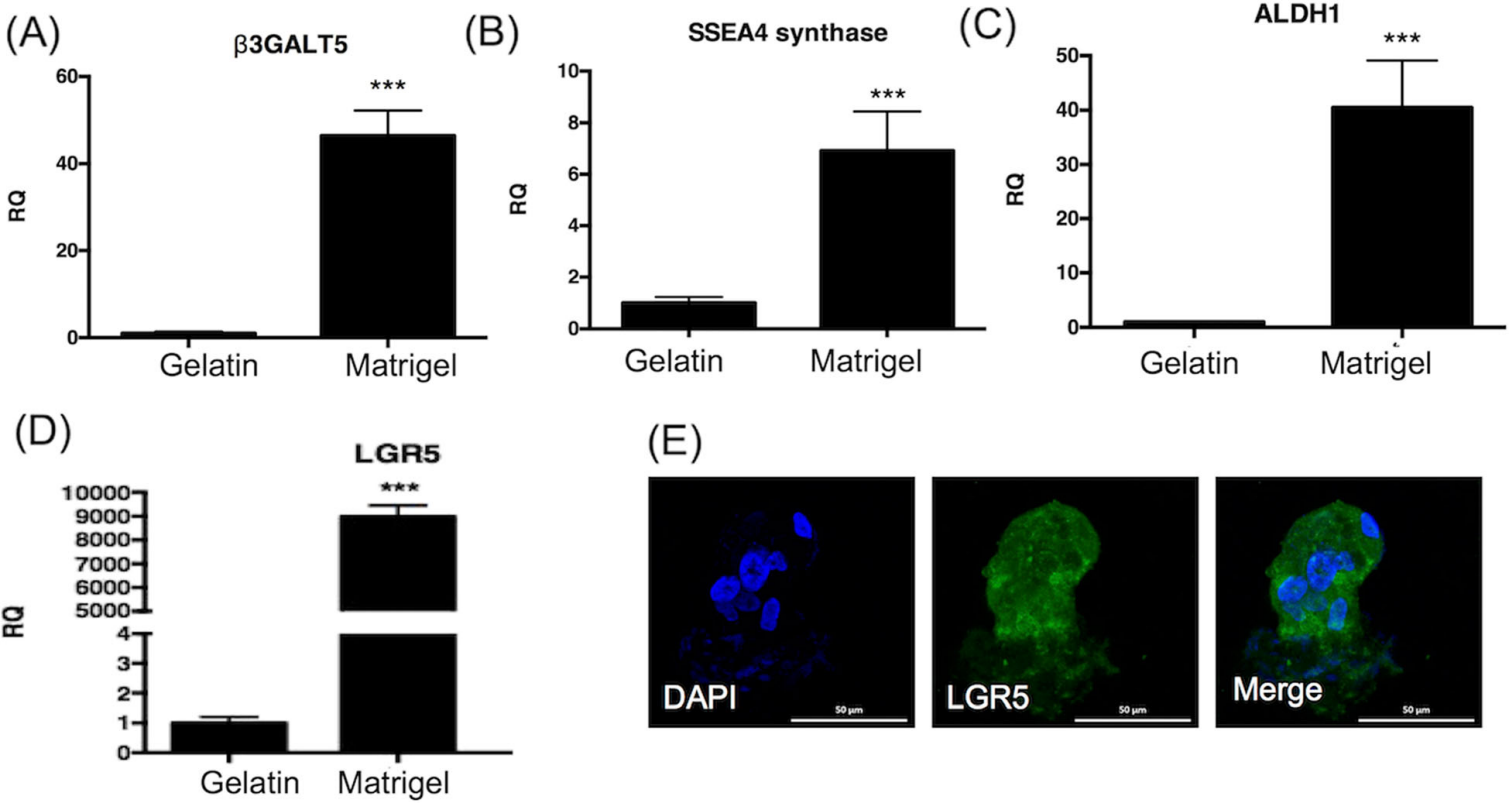
qRT-PCR of FTEC cultured on matrigel and gelatin. The qRT-PCR showed that FTECs cultured on Metrigel appeared to have an increased expression of (**a**) SSEA3 (*β3GALT5*), (**b**) SSEA4 (*SSEA4 synthase*), (**c**) ALDH1, and (**d**) LGR5 than those cultured on gelatin. (**e**) The FTEC colony revealed positive LGR5. DAPI (blue) staining represented cell nuclei. Scale bar = 50 μm

Gelatin, like Matrigel, can serve as a feeder for embryonic stem cell culturing [24] and show equal OCT4 expression. We compared the stem cell marker expression of FTEC cultured on gelatin vs. Matrigel. Compared to the gelatin matrix, Matrigel culture showed a much higher expression of SSEA3 (*β3GALT5*), SSEA4 (*SSEA4 synthase*), LGR5, and ALDH1 (*p* < 0.001, Fig. 7a-d). Among them LGR5 was 9000 folds increased and was also highly expressed in the colony formed on Matrigel (Fig. 7e). Taken together, Matrigel culture could greatly enhance the stemness of FTEC.

### FTEC formed organoids on Matrigel

**Fig. 8:**
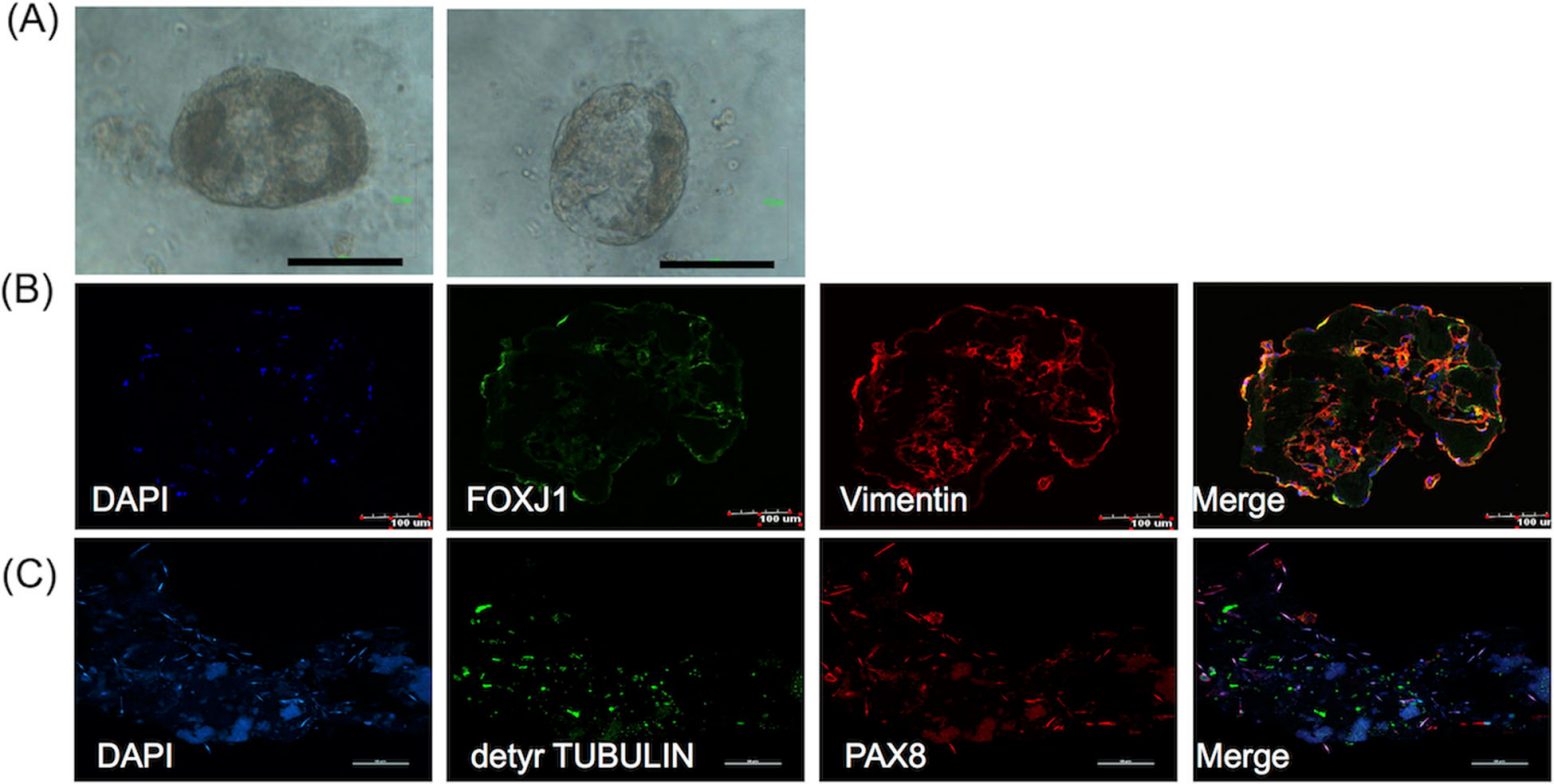
FTEC generated organoid after cultured on Matrigel for more than 21 days. (**a**) The morphology of organoid culture on matrigel. (**b**) The formed organoid was positive for vimentin (mesodermal marker) and FOXJ1 (ciliated cell marker). (**c**) The formed organoid was positive for detyrosinated TUBULIN (ciliated cell marker) and PAX8 (secretory cell marker)

After organoid culture (see M&M for culture condition) of 25,000 FTECs in each of the 6 -well dish precoated with Matrigel, organoid formed in xx% of the well after 21 days. The formed organoid showed a hollow-ball appearance (Fig. 8a). The organoid recaptured the structure of the fallopian tube and expressed the FTE markers including Vimentin, FOXJ1 (Fig. 8b), PAX8 and detyrosinated TUBULIN (Fig. 8c).

### A “contracted” organoid developed when FTEC were co-cultured with fallopian tube mesenchymal cells (FTMSC) and human umbilical vascular endothelial cells (HUVEC)

**Fig. 9:**
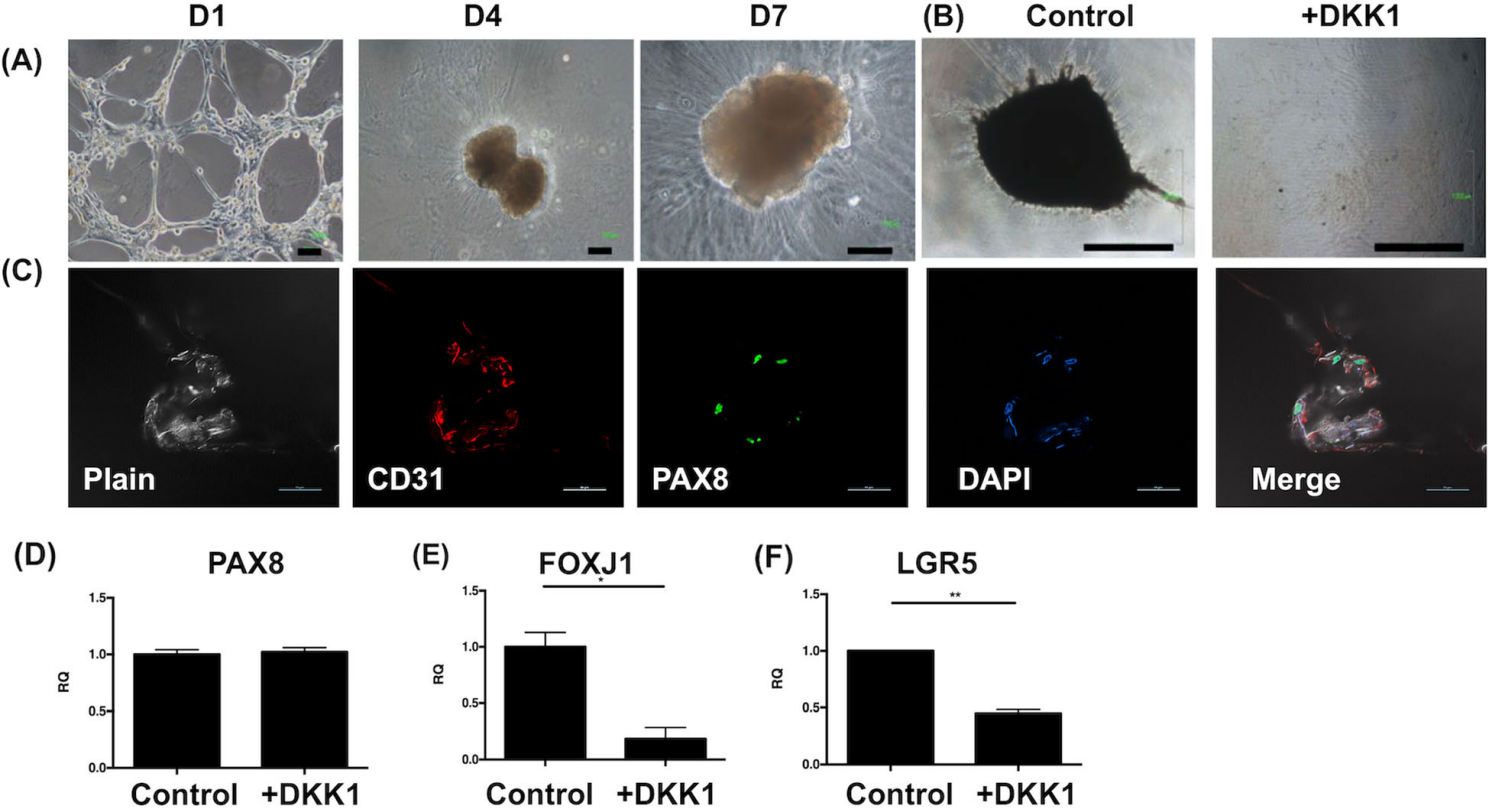
FTEC generated organoid-like mass after cocultured with human umbilical vein endothelial cells (HUVEC) and fallopian tube mesenchymal stem cells (FTMSC) and could be inhibited by DKK1. (**a**) Self-organization of three-dimensional FTEC co-cultured with HUVECs and FTMSCs on day 1, day 4, and day 7. (**b**) After adding DKK1 (Wnt signal inhibitor), no organoid was formed after 7 days. Scale bar = 100 μm. (**c**) The formed organoid positive staining for CD31 (endothelial marker) and PAX8 (secretory cell marker). Scale bar = 50 μm. The qRT-PCR showed that organoid with treating DKK1 appeared to have no influence on the expression of PAX8 (**d**) and a decrease in expressions of FOXJ1 (**e**) and LGR5 (**f**). *p < 0.05, **p < 0.01

In order to recapitulate the early organogenesis of the fallopian tube, FTECs were co-cultured with FTMSCs and HUVECs in Matrigel (see M&M for detail). At day 1, a unique structure of lattice appearance formed. After 4 days, the co-cultured cell population began to shrink toward the center of the dish forming a solid ball (Fig. 9a). In this culture condition, 100% of the loaded well-formed the organoid. The contracted organoid was diffusely positive for CD31 (endothelial cell) and focally positive for PAX8 (secretory cell) markers (Fig. 9c). Adding DKK1 to inhibit the Wnt signaling pathway blocked the organoid formation (Fig. 9b). Most of the cells exhibited fibroblastic morphology. DKK1 also partly decreased the expression of LGR5 and largely of FOXJ1, but did not affect the PAX8 expression (Fig. 9d). Above all, when co-cultured with HUVECs and FTMSCs, FTECs could generate “contracted” organoids that could be blocked by inhibiting the Wnt signaling pathway.

## Discussion

We successfully derived primary human FTECs from fallopian tube epithelium (FTE). In monolayer culture in a gelatin-coated dish, FTECs can be maintained in vitro up to P9. Two of the three lines expressed stem cell markers including LGR5, SSEA3, and SSEA4 in over 50% of the population and all the three lines highly expressed LGR5. They also expressed CD44, CD133, and ROR1 at P2. Approximately 14% of the population were ALDH-positive. These positive cells form more colonies of various sizes.

In the low-attachment culture with limited serum replacement growth factors, FTECs forms spheres with an efficiency of 4%. These spheres show characteristics of fallopian tube epithelium structure with ciliated cell and secretory differentiation. The presence of Matrigel much increased the stemness and colony formation of FTECs. After culturing on matrigel for 21 days, FTEC could form organoids and express stem cell-specific markers. In mimicking the organotypic culture, FTEC co-cultured with HUVEC and FTMSC formed a “contracted” organoid structure in high (100%) efficiency, and this organoid formation could be largely (100%) blocked by the Wnt inhibitor. Taken together, we demonstrated the high abundance of stem cell characteristics in primarily cultured human FTEC that are largely dependent on the Wnt signal. The stemness quickly lost in gelatin-coated matrix culture, and can be enhanced by Matrigel and maintained in the organoid culture.

A previous study has used a medium constituted of 25% of conditioned medium from fibroblasts to provide WNT3A and RSPO1 for fallopian tube organoid [25]. Another report used 100 ng/ml WNT3A and 600 ng/ml RSPO1 in the organoid culture [26]. In another study, a 10% homemade RSPO1 conditioned medium and 200 ng/ml WNT3A was used [27]. In our study, we used a less amount of WNT3A and RSPO1 (50 ng/ml each). The efficiency of organoid formation in our study may be further improved by increasing the concentrations of WNT3A and RSPO1.

Previous studies have shown using EPCAM+ cells could yield the highest efficiency of organoid forming [11, 26]. We also showed ALDH+ cells formed more colonies than ALDH- in this study. The other study using a 2D to 3D-switch culture to select the stem cell population [25]. Likewise, in our experiments, we cultured FTEC first in 2D gelatin dish, then transfer to 3D Matrigel culture. We observed LGR5 expression significantly increased after the switch.

In studying the transformation of the human fimbrial epithelial secretory cell, the presumed cell-of-origin of HGSOC, studies have used immortalized fimbrial epithelial cells for transformation [8, 28, 29] and showed clonal expansion and stemness activation on these p53/Rb-deficient FTEC. Although these studies clearly indicated fallopian tube epithelial cells follow the classical tumor evolution from precancerous to a cancerous development, they did not prove the very early step of tumor initiation, which is the transformation of the fallopian tube stem cell. This study demonstrated the abundant presence of stem cells in the fallopian tube fimbrial epithelium and provide the basis of the study of cancer initiation.

Regarding the specific marker panel for fallopian epithelial stem cells, one study indicated CD44, integrin α6, and EpCAM as the stem cell markers [11]; and the single epithelial cell could form organoids [25]. In another study where FTESCs are derived from isolated FTEC using a 3 T3 cell feeder culture [12], the resulted stem cell markers were PAX8+, FOXJ1-, and PAX2-. Another study shows distal fallopian tube quiescent cells could be the origin of stem cells differentiating into different glandular cells [13]. These proposed marker panels for FTESCs are not consistent. In the present study, we used common stem cell markers including LGR5 (FTESC maker) [30], SSEA3, SSEA4 (embryonic and cancer stem cell markers) [31] and ALDH (cancer stem cell marker) [32] to characterize the FTESC population in FTEC. SSEA3 and SSEA4 are not only embryonic stem cell markers but also markers for cancer stem cells [33, 34] and muscle cells [35]. We demonstrated that all three FTECs expressed LGR5 in a large proportion of the cell population. The other two markers were largely presented in two of the tree FTEC lines. It is worthy of future evaluation of this finding.

Markers of HGSOC stem cells may also be relevant to stem cells of FTEC. A previous study identified CD24, CD44, CD117, CD133, and ROR1 as ovarian cancer stem cells [17]. ALDEFLUOR™ and side population assays were also used for isolated ovarian cancer stem cells [17]. We found some CSC markers such as CD133, ROR1, and CD44 were expressed in half to almost all the populations in FTECs. With such a high representation, they may be hard to serve as a stemness marker in FTEC. The other markers, CD117 and CD24, were expressed in 10% or lower in the population, but are much less abundant than the well-recognized stemness marker LGR5. The link between these CSC markers to the development of FTEC to cancer needs further clarification.

Wnt and Notch signaling is important for the organoid growth and differentiation of FTEC in both human and mouse [25, 26]. Wnt signaling has also been tightly associated with cancer stem cells [36]. To regenerate a tissue such as a fallopian tube, stromal and vascular structures are crucial. Co-cultured with induced pluripotent stem cells with HUVEC and stromal cells has successfully regenerated the liver bud [22]. In this study, we found coculturing FTEC with HUVEC and FTMSC promotes the formation of organoids and could be blocked by Wnt inhibitor (DKK1). Our findings indicated that the Wnt signaling pathway may play an important role in FTEC organoid formation. We did not derive a fallopian tube structure by these three combine organoid cultures. Further trials with different proportions and sequences of cell combinational and culture conditions are needed for this regeneration by in vitro organoid culture.

## Conclusion

We successfully derived FTEC with the expression of the normal stem cell markers (LGR5, SSEA3, and SSEA4), cancer stem cell markers (CD24, CD44, CD117, ROR1, CD133, and ALDH), and characteristics of stem cell. Whether these stem cell markers, as well as Wnt signaling in the FTEC, would contribute to carcinogenesis related to the HGSC and ovarian cancers is worthy of future advanced study. These findings may provide new insight to investigate how FTEC may regenerate to induce cancer.

## Data Availability

All data generated or analyzed during the current study are included in this published article.
